# Electrohydraulic lithotripsy-assisted endoscopic retrograde appendicitis therapy for a giant appendiceal fecalith

**DOI:** 10.1055/a-2784-8284

**Published:** 2026-02-17

**Authors:** Zhi-Yuan Zou, Qin-Qin Yi, Lu Bai, Yan-Hui Tian, Li-Sheng Wang, De-Feng Li, Sheng-Gang Zhan

**Affiliations:** 1 12387 Department of Gastroenterology, Shenzhen Peopleʼs Hospital, Shenzhen, China

**Fig. 1:**
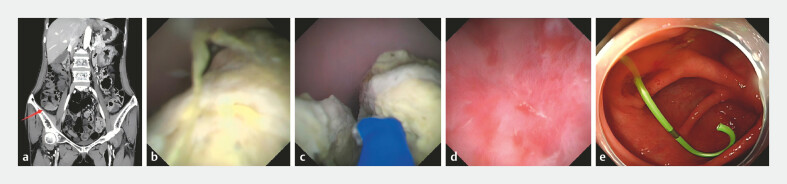
**a** An abdominal CT scan revealed appendicitis and a giant appendiceal fecalith. **b** A giant appendicolith was found by the EyeMax subscope in the appendiceal lumen. **c** Electrohydraulic lithotripsy (EHL) was performed to break the appendicolith. **d** The appendicolith was successfully removed. **e** A 5 Fr-5cm Single Pigtail Stent was successfully implanted. CT, computed tomography.

A 57-year-old woman complained of recurrent pain and tenderness in the right lower quadrant for 3 months. An abdominal computed tomographic scan revealed appendicitis and a giant appendiceal fecalith (2.5 × 3 cm; Fig. 1 **a** ). Therefore, the patient was admitted to remove the fecaliths by endoscopic retrograde appendicitis therapy (ERAT). The EyeMax subscope (Micro-Tech, Nanjing, China) was intubated into the appendiceal cavity, and found a giant appendicolith measuring approximately 3cm obstructing the appendiceal lumen ( Fig. 1 **b** ). Unfortunately, it was unsuccessful to remove the appendicolith using a basket. Subsequently, it was decided to perform electrohydraulic lithotripsy (EHL) to break the appendicolith and success the removal of them ( Fig. 1 **c, d** and Video 1 ). After this, a 5 Fr-5cm Single Pigtail Stent was successfully implanted ( Fig. 1 **e** ). Excitingly, the patient was recovered quickly from abdominal pain and was discharged 2 days later.

The procedure of electrohydraulic lithotripsy-assisted endoscopic retrograde appendicitis therapy for a giant appendiceal fecalith.Video 1

ERAT presents a pioneering strategy in the management of appendicoliths, as a groundbreaking minimally invasive procedure specifically conceived to alleviate appendiceal obstruction 1 2 . Appendicoliths are usually dimensionally less than 1 cm, with those larger than 2 cm designated as giant appendicoliths 3 . However, it is difficult to remove the giant appendicoliths by traditional strategies, such as basket, balloon, etc. In this case, electrohydraulic lithotripsy-assisted ERAT successfully removed a giant appendiceal fecalith, which maybe a promising approach in the administration of giant appendicoliths in the future.

Endoscopy_UCTN_Code_CCL_1AD_2AJ Endoscopy_UCTN_Code_TTT_1AQ_2AJ
